# Upper Gastrointestinal Bleeding From Aberrant Right Subclavian Artery-Esophageal Fistula

**DOI:** 10.31486/toj.20.0142

**Published:** 2021

**Authors:** Shira Peress, Wa’el Tuqan, Austin Thomas

**Affiliations:** ^1^ The University of Queensland Faculty of Medicine, Ochsner Clinical School, New Orleans, LA; ^2^ Department of Gastroenterology, Ochsner Clinic Foundation, New Orleans, LA

**Keywords:** *Aberrant right subclavian artery*, *aneurysm*, *esophageal fistula*, *gastrointestinal hemorrhage*

## Abstract

**Background:** The most common aortic arch abnormality is an aberrant right subclavian artery (ARSA). ARSA-esophageal fistula is a rare sequela that can present with a life-threatening upper gastrointestinal (GI) bleed.

**Case Report:** We report the case of an 88-year-old male who presented with signs of upper GI bleeding. Esophagogastroduodenoscopy demonstrated extrinsic compression of the upper third of the esophagus with ulceration. Imaging studies revealed ARSA posterior to the esophagus with pseudoaneurysm formation. These findings confirmed an upper GI bleed secondary to ARSA-esophageal fistula. The patient underwent prompt embolization of the ARSA pseudoaneurysm, followed a few days later by coil embolization of the ARSA pseudoaneurysm. Despite these interventions, the patient continued to have bleeding with anemia. He and his family opted to avoid any further interventions and instead pursued comfort care. The patient was discharged to hospice and died 3 months later.

**Conclusion:** ARSA-esophageal fistula is a rare but potentially lethal cause of upper GI bleeding. Initial signs and symptoms can be subtle, but the presence of a GI bleed requires immediate stabilization. Surgical interventions have been shown to have longer-lasting success, but endovascular repair may be an option for patients who are deemed unfit for surgery but still require prompt stabilization. Regardless of the intervention, mortality rates for ARSA-esophageal fistula are high.

## INTRODUCTION

Aberrant right subclavian artery (ARSA), or arteria lusoria, is the most common aortic arch abnormality, with a prevalence of 0.2% to 2.5%.^1,2^ ARSA is typically an incidental finding that seldom causes any symptoms.^3^ However, ARSA is prone to aneurysmal development that can remain asymptomatic or cause compressive symptoms, including shortness of breath and dysphagia lusoria secondary to extrinsic compression of the esophagus.^3^ The recurrent laryngeal nerve is also prone to a degree of constriction from ARSA, causing progressive hoarseness. An even rarer consequence is the development of ARSA-esophageal fistula that can present as a life-threatening upper gastrointestinal (GI) bleed, so prompt diagnosis and management are essential for patient survival.

## CASE REPORT

**Figure 1.**
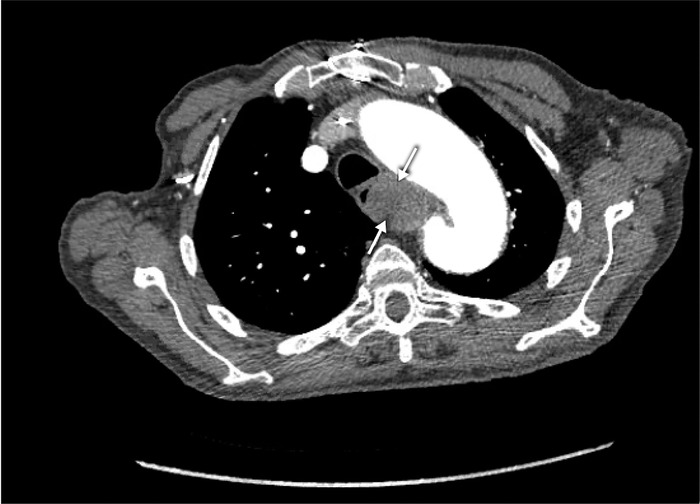
Computed tomography angiography of the chest demonstrates an ascending thoracic aortic aneurysm with saccular pseudoaneurysm formation. Arrows point to the aneurysm compressing the esophagus.

An 88-year-old male presented to the emergency department (ED) with a 1-day history of shortness of breath. He reported one episode of dark black, bloody stools 2 days prior. He also reported dysphagia for 6 months and hoarseness of voice for 1 week. He had an extensive cardiovascular history, including aortic stenosis status post transcatheter aortic valve replacement, thoracic aortic aneurysm (TAA), atrial fibrillation on warfarin, and congestive heart failure. A month and a half prior to his presentation, computed tomography (CT) angiography of the chest to evaluate the TAA demonstrated an ascending TAA and saccular pseudoaneurysm formation from an ARSA, resulting in marked mass effect and displacement of the esophagus (Figure 1). Bilateral carotid subclavian bypass was recommended; however, the patient declined surgery at the time.

**Figure 2.**
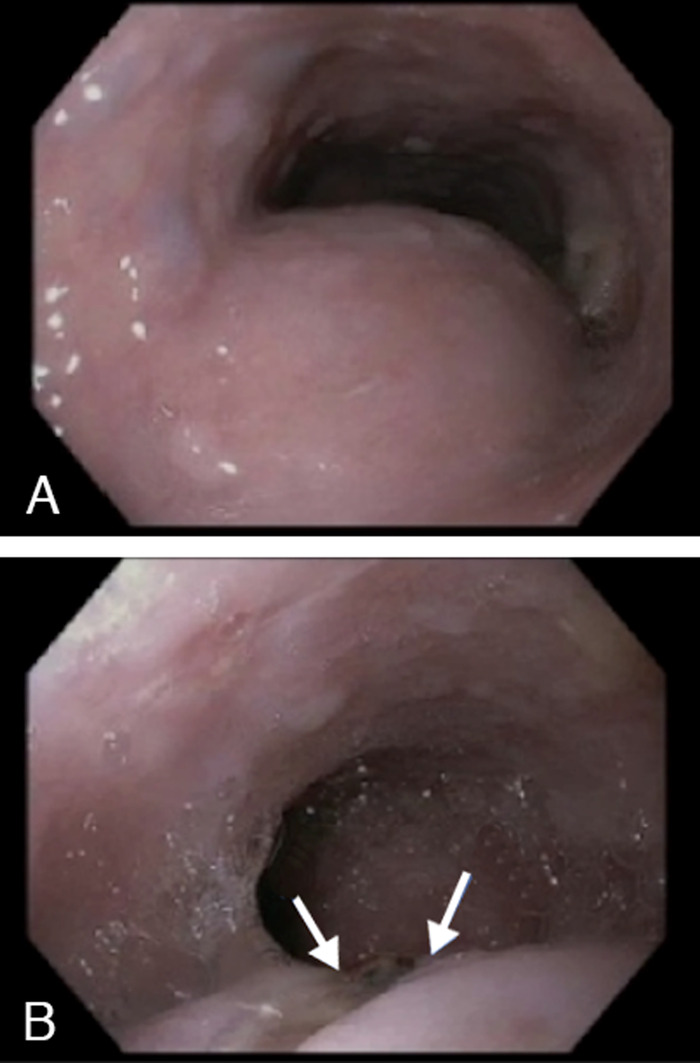
Esophagogastroduodenoscopy demonstrates (A) external compression into the lumen of the esophagus and (B) the inferior part of the compression with ulceration (arrows) and possible visible vessel.

On presentation to the ED, he was hemodynamically stable. Laboratory workup revealed hemoglobin of 6.5 g/dL from a baseline of 11.0 g/dL. Prothrombin time was 17.1 seconds, with an international normalized ratio (INR) of 1.8. Following initial resuscitation with 2 units of packed red blood cells and intravenous pantoprazole, esophagogastroduodenoscopy (EGD) was performed and demonstrated extrinsic compression of the upper third of the esophagus, with associated areas of ulceration and visible underlying vasculature (Figure 2). EGD findings were concerning for development of ARSA-esophageal fistula and the potential for subsequent bleeding. Vascular surgery performed urgent embolization of the ARSA using Amplatzer plugs.

**Figure 3.**
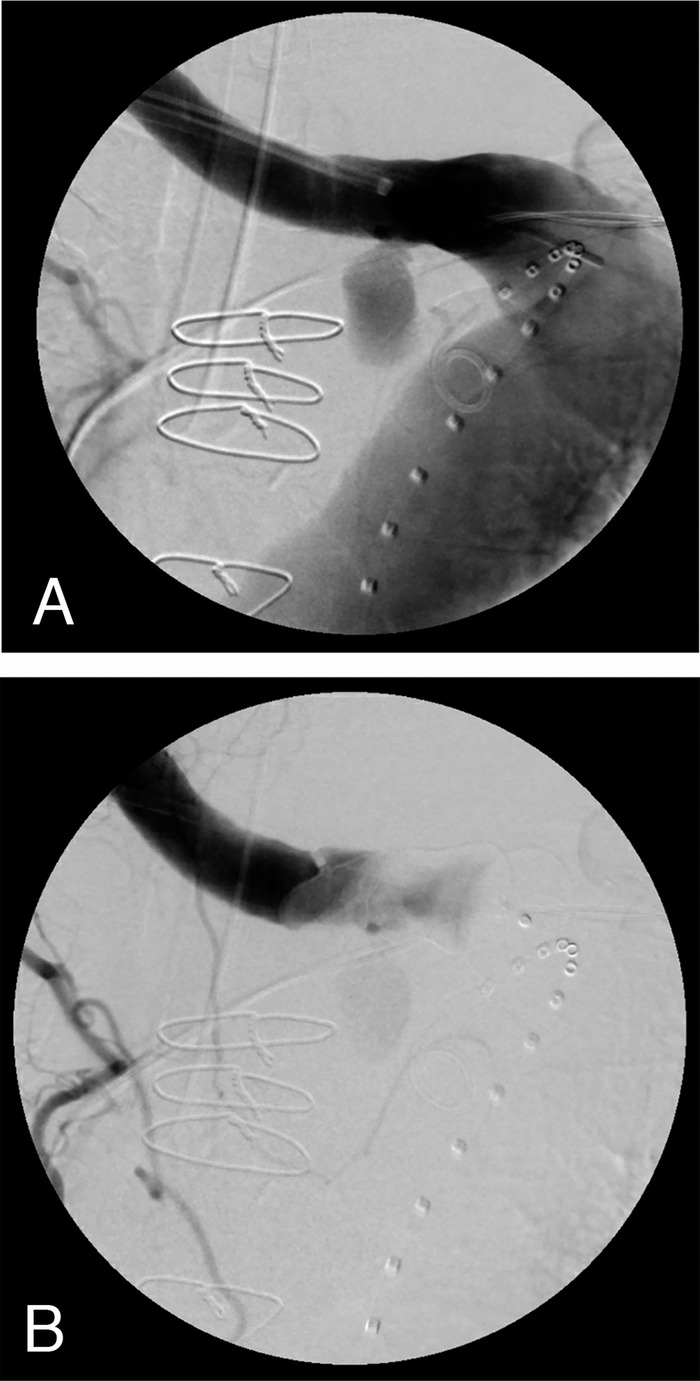
Interventional radiology imaging demonstrates (A) precoiled pseudoaneurysm and (B) postcoiled pseudoaneurysm.

The patient initially did well and had no further signs of bleeding. He was discharged 2 days after the procedure with hemoglobin of 7.7 g/dL and INR of 1.2. One day after discharge, he presented again to the ED with worsening fatigue, weakness, and shortness of breath. He had 2 additional episodes of melena and a drop in hemoglobin to 6.5 g/dL. He was readmitted, and repeat CT angiography demonstrated a new bleed within the pseudoaneurysm sac. In conjunction with vascular surgery, interventional radiology performed a coil embolization of the ARSA pseudoaneurysm (Figure 3).

Despite these interventions, the patient continued to have ongoing signs of bleeding with anemia. He required multiple blood transfusions for stabilization, ultimately receiving 5 units of packed red blood cells that helped improve his hemoglobin to 9.2 g/dL and hematocrit to 28.5%. After multidisciplinary team discussions, the patient and his family opted to avoid any further workup or surgical interventions and instead pursued comfort care. He was discharged to hospice 4 days after being readmitted and died 3 months later.

## DISCUSSION

**Figure 4.**
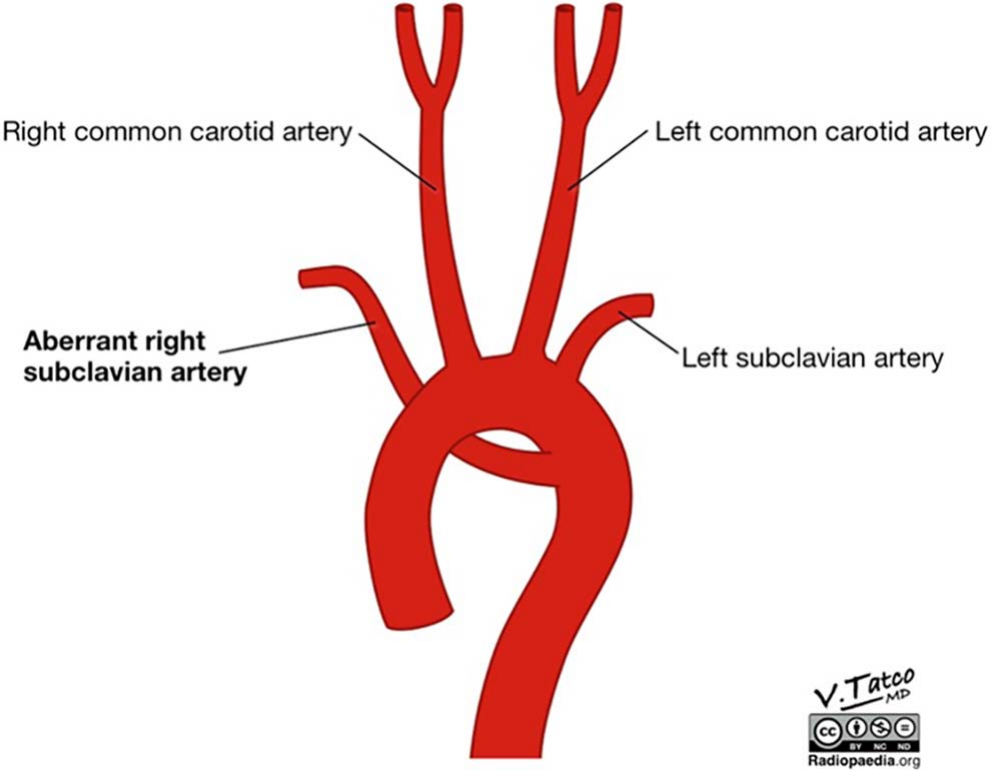
**Schematic representation of aberrant right subclavian artery.** (Image courtesy of Dr Vincent Tatco, Radiopaedia.org, rID: 52193 radiopaedia.org/cases/development-of-aberrant-right-subclavian-artery-illustration)

When ARSA is present, the right subclavian artery branches distal to the left subclavian artery and serves as the fourth branch of the left-sided aortic arch (Figure 4). From there, the artery travels posterior to the esophagus in 80% of cases but can also be found between the trachea and esophagus in 15% of cases and anterior to the trachea in 5% of cases.^4^ ARSA has been reported to have a female predominance as high as 3:1, although other studies suggest no significant sex discrepancy.^5^ ARSA can be associated with other medical conditions and is seen in up to 25% of patients with esophageal atresia and in 16% to 39% of patients with Down syndrome.^6,7^

Despite its prevalence in the general population, ARSA is frequently asymptomatic and discovered incidentally on imaging. However, ARSA can present with dysphagia secondary to esophageal compression, a phenomenon known as dysphagia lusoria.^8^ Depending on the exact location, ARSA can cause some degree of compression of the recurrent laryngeal nerve, resulting in hoarseness, a condition known as Ortner syndrome.^9^ ARSA can also cause compression of the trachea, resulting in shortness of breath. With advancing age, ARSA is prone to aneurysmal development or sclerosis.^3^ The late presentation of dysphagia in our elderly patient is not uncommon, as dysphagia is more frequently seen in the elderly compared to younger patients with ARSA and can be attributed to decreased flexibility of the esophagus or increasing compression from the developing aneurysm. Some degree of atherosclerosis contributing to rigidity of the ARSA is also possible.^10^

In our review of literature, we found 35 reported cases of ARSA-esophageal fistula.^1,11-43^ Of the 35 cases, 33 cases are included in our summary table, while 2 cases were excluded due to inability to access the primary source or the source being in a language other than English (Table).^13,20^ Six cases were in the pediatric population, with patient ages ranging from 5 months to 11 years.^22,28,30,34,37^ Although diagnosis is typically made with CT imaging, endoscopic visualization may be the initial diagnostic test to exclude other causes, particularly in patients who are hemodynamically stable at the time of presentation. The most common etiology for development of ARSA-esophageal fistula appeared to be secondary to compression, friction, or pressure necrosis from recent instrumentation with an endotracheal tube, nasogastric tube, or tracheostomy tube, seen in 14 of the 33 summarized cases.^14,15,17,19,21,22,24,27,29,31,38-41^ Fistula development from prior placement of esophageal stent was noted in 5 cases.^34,36,42,43^ Other reported causes for fistula formation included placement of a salivary bypass tube and a possible consequence of gastric pull-up surgery.^1,26^

**Table.** Summary of Reported Cases of Aberrant Right Subclavian Artery-Esophageal Fistula

Study	Age, Sex	Medical History	Etiology	Treatment	Outcome
Lynn, 1969^11^	57, M	Atherosclerosis, hypertension, angina	ARSA aneurysm	Surgery	Fatal
Reynes et al, 1976^12^	72, F	Mediastinal mass	ARSA aneurysm	None	Fatal
Livesay et al, 1982^14^	25, M	Motor vehicle accident with traumatic head injury	Endotracheal tube, nasogastric tube, and tracheostomy	Balloon tamponade, surgery	Fatal
Belkin et al, 1984^15^	27, M	Head and neck squamous cell carcinoma	Nasogastric tube	Balloon tamponade, surgery	Fatal
Edwards et al, 1984^16^	81, M	Mass in right upper hemithorax (tortuous innominate artery)	ARSA aneurysm	Surgery	Fatal
Gossot et al, 1985^17^	72, F	Type I aortic dissection with endoluminal prosthesis, giant cell arteritis	Endotracheal tube, nasogastric tube, and tracheostomy	None	Fatal
Kullnig, 1989^18^	66, M	Hypertension	ARSA aneurysm	None	Fatal
Stone et al, 1990^19^	72, M	Smoker, status post cardiopulmonary bypass surgery	ARSA aneurysm, tracheostomy	None	Fatal
Hirakata et al, 1991^21^	55, M	Status post subtotal esophagectomy for esophageal cancer	Nasogastric tube, radiation arteritis, and other surgical trauma	Balloon tamponade	Survived
Miller et al, 1996^22^	11, F	Status post craniotomy for intracerebral hemorrhage	Endotracheal tube, nasogastric tube	Balloon tamponade, surgery	Survived
Singha et al, 1998^23^	82, M	N/R	ARSA aneurysm	None	Fatal
Feugier et al, 2002^24^	24, M	Polytrauma, burns, alcohol abuse	Nasogastric tube and tracheotomy	Balloon tamponade, surgery	Survived
Lehmann et al, 2006^25^	78, M	N/R	ARSA aneurysm	Balloon tamponade	Fatal
Millar et al, 2007^26^	57, M	Status post esophagectomy with gastric pull-up for esophageal cancer	Pressure from gastric pull-up vs foreign body	Surgery	Fatal (survived initial hemorrhage but died 18 days later from additional bleed)
Inman et al, 2008^1^	63, M	Supraglottic squamous cell carcinoma	Salivary bypass tube	Endovascular repair, balloon tamponade	Fatal
Magagna et al, 2008^27^	73, F	Status post laryngectomy and tracheostomy for laryngeal carcinoma	Tracheostomy	Endovascular repair, balloon tamponade	Survived
Fuentes et al, 2010^28^	3, F	Esophageal atresia type III	Esophageal prosthesis	Endovascular repair, balloon tamponade, surgery	Survived
Chapman et al, 2010^29^	34, F	Motor vehicle accident with trauma	Endotracheal tube, nasogastric tube, and tracheostomy	Endovascular repair, balloon tamponade, surgery	Fatal
Situma et al, 2011^30^	5 months, F	Esophageal atresia with distal fistula	Status post colonic esophageal grafting	Surgery	Survived
Jain et al, 2012^31^	57, F	Status post cardiopulmonary bypass surgery for scimitar syndrome	Endotracheal tube, nasogastric tube, and tracheostomy	Endovascular repair, balloon tamponade, surgery	Survived
Pop et al, 2012^32^	67, M	Status post transhiatal esophagectomy for esophageal cancer, status post pneumonectomy for lung cancer, neuroendocrine liver cancer	Damage to arterial wall from cervical sepsis and/or staples from gastric tubing	Endovascular repair, balloon tamponade	Fatal
Takahashi et al, 2013^33^	63, M	N/R	Infected ARSA aneurysm	Balloon tamponade, surgery	Survived
Lo et al, 2013^34^	16 months, N/R	Esophageal atresia with distal fistula	Esophageal and Polyflex airway stent	Endovascular repair, Bougienage tamponade	Fatal
	18 months, N/R	Esophageal atresia, duodenal atresia	Esophageal stent	Surgery, hydrostatic dilator	Survived
Morisaki et al, 2014^35^	74, F	Rheumatic arthritis	ARSA aneurysm	Endovascular repair, balloon tamponade, surgery	Fatal
Hosn et al, 2014^36^	29, F	Sleeve gastrectomy	Esophageal stent	Endovascular repair, balloon tamponade, surgery	Survived
Joynt and Grifka, 2015^37^	17 months, F	N/R	Spontaneous development of fistula	Balloon tamponade, surgery	Survived
Watanabe et al, 2015^39^	55, M	Intracranial hemorrhage	Nasogastric tube and tracheostomy	None	Fatal
Oliveira et al, 2016^38^	20, M	Motor vehicle accident with polytrauma	Endotracheal tube and nasogastric tube	Surgery	Survived
Kudose et al, 2017^40^	20, F	VATER (vertebral, anal, tracheal, esophageal, and renal) association, primary pulmonary hypertension, diabetes mellitus, atrial septal defect, status post 3 lung transplants	Tracheostomy	None	Fatal
Shires and Rohrer, 2018^41^	44*,* F	Gastroesophageal reflux disease, hypertension, pneumonia	Nasogastric tube, endotracheal tube, and tracheostomy	Endovascular repair	Fatal
Zheng et al, 2019^42^	67, M	Esophageal and laryngeal cancer, hypertension	Esophageal stent and pseudoaneurysm	Endovascular repair	Fatal
Merlo et al, 2020^43^	29, F	Tracheoesophageal fistula, ventriculoperitoneal shunt for hydrocephalus	Esophageal stent	Endovascular repair, surgery	Survived
Present case, 2021	88, M	Hypertension, transcatheter aortic valve replacement, thoracic aortic aneurysm	ARSA aneurysm	Endovascular repair	Fatal (survived initial event, but died 3 months after discharge from recurrent bleeds)

Note: Age is given in years unless otherwise indicated.

ARSA, aberrant right subclavian artery; F, female; M, male; N/R, not reported.

Fistula formation attributable to ARSA aneurysm with resultant GI bleeding is rare. Only 10 other cases attributed to this etiology have been reported.^11,12,16,18,19,23,25,33,35,42^ Only 1 patient with upper GI bleed secondary to ARSA aneurysm survived all bleeding episodes, and the patient required balloon tamponade with surgery to replace the aorta.^33^ Our patient survived the initial bleeding event, but died 3 months later, as he declined definitive surgical intervention to stop the recurrent bleeding.

Bleeding from ARSA-esophageal fistula is life-threatening, with a high mortality rate at initial presentation, regardless of etiology. Of the 33 summarized cases, thirteen cases survived all episodes of bleeding (39%).^21,22,24,27,28,30,31,33,34,36-38,43^ The severity of bleeding, in the setting of several comorbid conditions, likely contributed to the high mortality rate.

Opinions conflict regarding acute treatment for bleeding from ARSA-esophageal fistula secondary to aneurysm, with the historic approach being excision with reconstruction of the aorta and esophagus.^33^ A more recent approach (2000) is placement of an endovascular stent, but success with this approach is questionable because of the rates of infection that may result in the need for further surgery.^44^ Reported cases in adults involved treatment with surgery alone in 5 cases,^11,16,26,30,38^ endovascular repair alone in 2 cases,^41,42^ and a combination of endovascular and surgical intervention in 6 cases.^28,29,31,35,36,43^ Balloon tamponade was performed in 16 cases,^1,14,15,21,22,24,25,27-29,31-33,35-37^ with 3 of those 16 performed solely with endovascular repair.^1,27,32^ Six of the 11 patients who underwent either surgery alone or surgery with endovascular repair survived (55%),^28,30,31,36,38,43^ whereas only 1 of the 5 cases of endovascular repair with or without balloon tamponade survived (20%).^27^ Although the higher percentage of survival among patients who had surgery with or without endovascular repair compared to the 5 patients who underwent endovascular repair without surgery might suggest a better outcome with surgery, this discrepancy is likely confounded by patient factors and comorbidities for which surgical intervention is usually deferred, as well as the relatively low number of patient cases reported to have been treated with endovascular repair without surgery.

## CONCLUSION

ARSA-esophageal fistula is an uncommon and potentially fatal cause of GI bleeding. Patients with known ARSA aneurysm should elicit a high index of suspicion, particularly those who present with concomitant worsening dysphagia. Given the high mortality with active bleeding, endovascular repair is a potential alternative for patients who are not suitable for surgery because of their comorbidities or high surgical risk. However, even with intervention, mortality remains high.

## Notes

*This article meets the Accreditation Council for Graduate Medical Education and the American Board of Medical Specialties Maintenance of Certification competencies for Patient Care and Medical Knowledge.*
